# Ultrasound Evaluation and Treatment of Posterior Medial Antebrachial Cutaneous Nerve Injury Following Cubital Tunnel Release

**DOI:** 10.3390/diagnostics16070960

**Published:** 2026-03-24

**Authors:** Wei-Ting Wu, Ke-Vin Chang, Levent Özçakar

**Affiliations:** 1 Department of Physical Medicine and Rehabilitation, Community and Geriatric Research Center, National Taiwan University Hospital, Bei-Hu Branch, Taipei 108206, Taiwan; wwtaustin@yahoo.com.tw; 2 Department of Physical Medicine and Rehabilitation, National Taiwan University College of Medicine, Taipei 100233, Taiwan; 3 Center for Regional Anesthesia and Pain Medicine, Wang-Fang Hospital, Taipei Medical University, Taipei 110301, Taiwan; 4 Department of Physical and Rehabilitation Medicine, Hacettepe University Medical School, Ankara 06100, Turkey; lozcakar@yahoo.com

**Keywords:** ultrasonography, injection, medial antebrachial cutaneous nerve, entrapment, hydrodissection

## Abstract

This case highlights the diagnostic value of high-resolution ultrasonography in identifying postoperative injury of the posterior branch of the medial antebrachial cutaneous nerve (MABCN) following cubital tunnel surgery. A 45-year-old man developed localized pain, warmth, and paresthesia extending from the medial epicondyle to the proximal anterior forearm one month after ulnar nerve decompression and anterior transposition. Physical examination revealed focal allodynia and a positive Tinel sign. Because previous surgery may substantially alter the anatomical location of the surrounding nerves, electrodiagnostic localization can be technically challenging and less reliable. Ultrasonography therefore played a key diagnostic role. The images demonstrated the normal sonoanatomy of the MABCN and its anatomical relationship with the basilic vein and ulnar nerve, followed by pathologic findings of focal enlargement of the posterior branch adjacent to postoperative scar tissue. These imaging features, together with a positive sonographic Tinel sign, supported the diagnosis of localized nerve irritation. Ultrasound-guided hydrodissection using 5% dextrose and methylcobalamin resulted in marked clinical improvement, with the Visual Analog Scale pain score decreasing from 9 to 2. This case is particularly illustrative because postoperative injury of the MABCN may mimic recurrent cubital tunnel syndrome yet typically produces purely sensory symptoms confined to the medial elbow region. Targeted ultrasonographic evaluation can reveal subtle postoperative nerve pathology and guide effective ultrasound-guided intervention.

**Figure 1:**
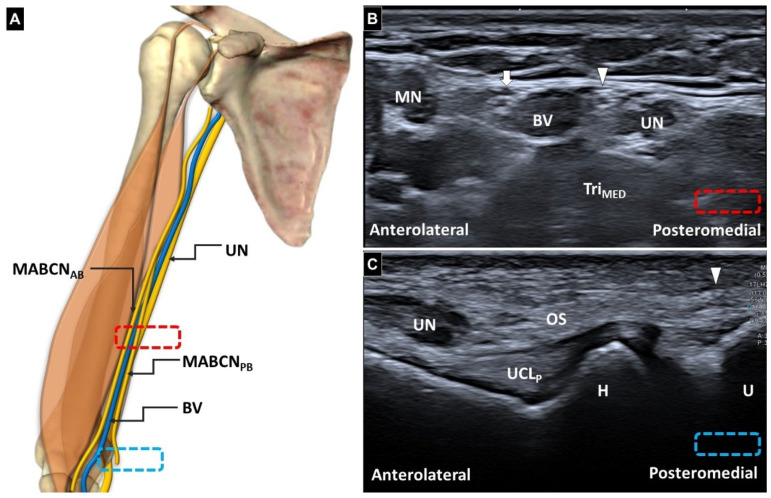
Illustration of the medial antebrachial cutaneous nerve in the arm (**A**). Short-axis ultrasonographic images of a normal medial antebrachial cutaneous nerve at the mid-arm (**B**) and cubital tunnel (**C**). Red and blue dashed square: locations of the transducer; White arrow and MABCN_AB_: anterior branch of the medial antebrachial cutaneous nerve; white arrowhead and MABCN_PB_: posterior branch of the medial antebrachial cutaneous nerve; UN: ulnar nerve; MN: median nerve; BV: basilic vein; OS: Osborne’s ligament; H: humerus; U: ulna; UCL_P_: posterior bundle of the ulnar collateral ligament; Tri_MED_: medial head of the triceps brachii.

**Figure 2:**
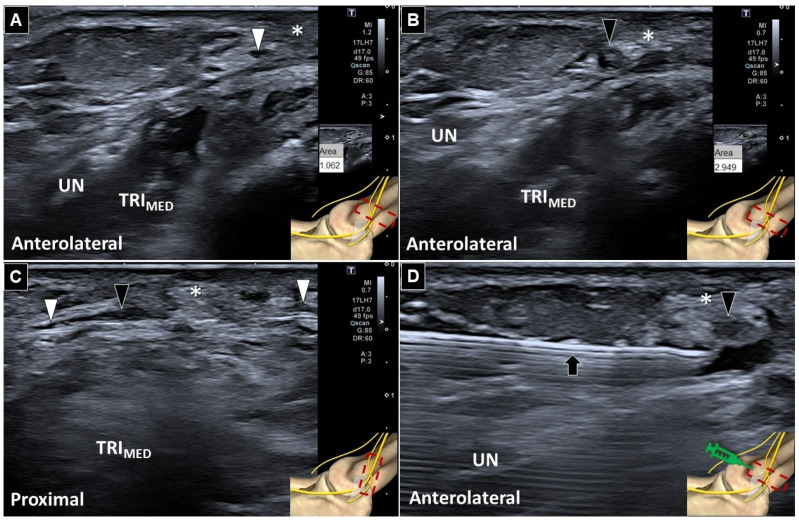
Ultrasonography demonstrates that the normal segment of the posterior branch of the medial antebrachial cutaneous nerve (white arrowheads) was located medial to the ulnar nerve, with a cross-sectional area of 1.062 mm^2^ (**A**). In contrast, the affected nerve showed a cross-sectional area of 2.949 mm^2^ on short-axis (**B**) and long-axis (**C**) views. The affected nerve appeared swollen (black arrowheads) and entrapped by the adjacent scar tissue (asterisk). Ultrasound-guided hydrodissection was subsequently performed using an in-plane approach, with a 25-gauge needle advanced under a short-axis view of the nerve to release the nerve from the surrounding scar adhesions. An anechoic injectate was simultaneously distributed circumferentially around the nerve (**D**). This image was obtained during the injection. Red dashed square: location of the transducer. Black arrow: needle; UN: ulnar nerve; TRI_MED_: medial head of the triceps brachii. The ultrasonographic images were obtained from a 45-year-old man who had undergone surgical release for cubital tunnel syndrome—with decompression extending approximately 6 cm proximal and distal to the medial epicondyle. After release of the humeral and ulnar heads of the flexor carpi ulnaris and neurolysis of its motor branch, a semicircular fascial flap had been developed from the flexor–pronator mass, followed by subfascial anterior transposition of the ulnar nerve with creation of a 1-cm-wide trough. After one month of immobilization followed by activities of daily living training, the patient developed painful warmth, swelling, and tingling sensations extending from the medial epicondyle to the proximal anterior forearm. The patient presented with purely sensory abnormalities and pain localized to the region extending from the medial epicondyle to the proximal anterior forearm. On physical examination, palpation over the symptomatic area elicited marked allodynia and a well-defined Tinel sign. Furthermore, because the patient had previously undergone ulnar nerve transposition surgery, the anatomical position of the surrounding nerves was likely substantially altered. Given the clearly localized clinical presentation and the anticipated limitations in electrodiagnostic localization under these conditions, electrodiagnostic studies (EMG/NCV) were not performed. Musculoskeletal ultrasound examination was performed using an Aplio i700 ultrasound system equipped with a high-frequency linear array transducer (17LHZ, 5–17 MHz; Canon Medical Systems Corporation, Otawara, Japan). Initially, to delineate the normal sonoanatomy (Figure 1A), the transducer was placed over the mid-arm of the unaffected side, between the median and ulnar nerves. The anterior and posterior branches of the medial antebrachial cutaneous nerve (MABCN) were identified adjacent to the basilic vein (Figure 1B) [1]. The posterior branch was then traced distally alongside the ulnar nerve toward the cubital tunnel. Whereas the ulnar nerve entered the tunnel beneath Osborne’s ligament, the posterior branch of the MABCN remained superficial and did not pass through the tunnel (Figure 1C) [2]. Compared with the proximal segment of the posterior branch of the medial antebrachial cutaneous nerve, the involved nerve demonstrated focal enlargement adjacent to the scar tissue (Figure 2), accompanied by a positive sonographic Tinel sign. Based on the diagnosis of nerve irritation, ultrasound-guided hydrodissection was performed (Figure 2D) using 5 mL of 5% dextrose solution combined with 1 mL of methylcobalamin (500 μg), resulting in subsequent symptom resolution. Methylcobalamin was selected as an adjunctive injectate based on its potential neurotrophic and analgesic effects, although its use in hydrodissection is not yet standardized. Experimental studies have demonstrated that vitamin B12 promotes peripheral nerve regeneration by enhancing Schwann cell proliferation and upregulating neurotrophic factors such as brain-derived neurotrophic factor (BDNF), thereby facilitating functional recovery after nerve injury [3]. In addition, clinical and preclinical evidence suggests that methylcobalamin may alleviate neuropathic pain through multiple mechanisms, including support of myelin synthesis, modulation of inflammatory pathways, and improvement of nerve conduction [4]. Given its favorable safety profile and potential synergistic effects in nerve healing, methylcobalamin was used in this case as an adjunct to hydrodissection. The intervention was performed using an in-plane approach under guidance, with a 25-gauge needle advanced along the short-axis view of the nerve. No procedural complications were observed. The patient’s pain severity improved substantially following treatment, with the Visual Analog Scale score decreasing from 9 before the procedure to 2 after the intervention. At the one-month follow-up, the patient reported no recurrence of pain. On physical examination, localized tenderness and the Tinel sign were no longer present. Follow-up ultrasonography demonstrated that the previously identified entrapment site had become less conspicuous. The MABCN is a purely sensory nerve arising predominantly from the C8–T1 fibers of the medial cord of the brachial plexus [5]. It descends along the medial aspect of the arm between the brachialis and triceps brachii muscles, traverses the basilic hiatus in close association with the basilic vein, and subsequently becomes subcutaneous. Between the mid-arm and distal arm, the MABCN typically bifurcates into anterior and posterior branches [6]. The former usually crosses the elbow anteriorly between the long head of the biceps tendon and the medial epicondyle, providing sensory innervation to the anteromedial forearm and antecubital fossa. As such, it is susceptible to injury during venous puncture [7]. In contrast, the posterior branch courses posterior to the medial epicondyle and supplies sensation to the posteromedial elbow, olecranon region, and posteromedial forearm. Several anatomical and procedural factors predispose the MABCN to iatrogenic injury during cubital tunnel surgery [8,9,10,11], elbow arthroscopy [12], open elbow procedures [13], and injections for medial epicondylitis [14]. Its superficial course and small caliber hinder intraoperative identification, while the posterior branch frequently lies between the medial epicondyle and basilic vein—directly within the conventional medial elbow incision zone. Moreover, substantial variability in branching patterns implies that even short or minimally invasive incisions do not reliably protect the nerve from inadvertent injury. In particular, longitudinal incisions made parallel to the ulnar nerve may increase the likelihood of transecting the posterior branches of the MABCN. Clinically, injury to the posterior branches of the MABCN may result in persistent medial elbow pain, dysesthesia, hypoesthesia, or painful neuroma formation. These manifestations may mimic recurrent cubital tunnel syndrome and can adversely affect postoperative outcomes, potentially leading to diagnostic confusion and unnecessary revision surgery. Injury to the posterior branch of the MABCN typically produces purely sensory symptoms confined to a limited region a few centimeters proximal and distal to the medial epicondyle, including persistent pain, paresthesia, and numbness. In contrast, recurrent cubital tunnel syndrome is more commonly associated with motor deficits and muscle atrophy, and the distribution of symptoms is usually broader and more distal, potentially extending from the forearm to the fingers. In patients who have undergone anterior transposition of the ulnar nerve, the symptomatic region may shift anteriorly and appear closer to the pronator teres muscle. This case illustrates a potential diagnostic pitfall in which persistent medial elbow pain following cubital tunnel release may be misinterpreted as recurrent ulnar neuropathy. In this patient, high-resolution ultrasonography demonstrated that the symptoms originated from focal enlargement and irritation of the posterior branch of the MABCN adjacent to postoperative scar tissue, thereby clarifying the true etiology. In addition, this case provides a valuable ultrasonographic demonstration of the posterior branch of the MABCN, a structure that is often difficult to identify because of its small caliber, superficial course, and anatomical variability. Through systematic proximal-to-distal tracing and side-to-side comparison, the nerve and its relationship to the basilic vein and the ulnar nerve were clearly delineated, enabling precise localization of the entrapment site. Furthermore, this report highlights the therapeutic role of ultrasonography. Targeted ultrasound-guided hydrodissection of the affected nerve resulted in resolution of the patient’s symptoms. This imaging-guided approach underscores the dual diagnostic and interventional utility of ultrasonography in the evaluation and management of postoperative neuropathic pain. To mitigate these risks, several preventive strategies have been proposed. A modified curved medial skin incision that deviates anteriorly toward the medial intermuscular septum has been shown to significantly reduce the likelihood of nerve transection. In addition, antegrade subcutaneous dissection—initiated proximally to identify the main posterior branch before tracing its distal branches—facilitates systematic preservation of the nerve. Consistent use of anatomical landmarks, particularly the medial epicondyle and the basilic vein, further aids early nerve identification and guides safe dissection planes. High-resolution ultrasonography has emerged as a valuable adjunct for both preoperative planning and postoperative evaluation of the MABCN. It also facilitates accurate postoperative evaluation by mitigating diagnostic difficulties arising from surgically altered anatomy and compression by postoperative scar tissue. On ultrasound, the nerve typically appears as a small hyperechoic fascicular structure within the subcutaneous layer [15]. From a practical standpoint, examination should begin proximally at the mid-arm, followed by distal tracing as the nerve bifurcates. Short-axis imaging combined with side-to-side comparison enhances detection, while color Doppler imaging aids in differentiating the nerve from adjacent superficial veins. Dynamic assessment during elbow flexion may further clarify nerve displacement relative to surgical landmarks. In addition, because the ultrasound beam may generate thermal and cavitation effects, stimulation of an injured nerve can occasionally elicit a positive sonographic Tinel sign. During examination, the patient should be positioned in a neutral and relaxed posture while avoiding excessive transducer pressure over the nerve. If necessary, gentle tapping with the probe over the suspected site may help accentuate the symptomatic response. In the postoperative setting, ultrasonography is particularly useful for identifying neuroma formation, which typically appears as a hypoechoic mass, or for detecting entrapment of the MABCN within postoperative scar tissue. In cases of entrapment, the affected nerve segment appears narrowed, while the proximal and distal portions may demonstrate fusiform swelling on long-axis views. The pathologies are frequently missed by electrodiagnostic studies. The MABCN, particularly its posterior branch, is prone to injury during cubital tunnel surgery because of its superficial course, anatomical variability, and proximity to standard surgical landmarks. Thorough anatomical understanding, modified incision techniques, adjunctive high-resolution ultrasonography, and ultrasound-guided interventions may help reduce iatrogenic injury as well as postoperative sensory morbidity.

## Data Availability

The original contributions presented in the study are included in the article, further inquiries can be directed to the corresponding author.
